# Ectopic γ-catenin Expression Partially Mimics the Effects of Stabilized β-catenin on Embryonic Stem Cell Differentiation

**DOI:** 10.1371/journal.pone.0065320

**Published:** 2013-05-27

**Authors:** Sujeivan Mahendram, Kevin F. Kelly, Sabrina Paez-Parent, Sharmeen Mahmood, Enio Polena, Austin J. Cooney, Bradley W. Doble

**Affiliations:** 1 Stem Cell and Cancer Research Institute, McMaster University, Hamilton, Ontario, Canada; 2 Department of Biochemistry and Biomedical Sciences, McMaster University, Hamilton, Ontario, Canada; 3 Department of Molecular and Cellular Biology, Baylor College of Medicine, Houston, Texas, United States of America; Northwestern University Feinberg School of Medicine, United States of America

## Abstract

β-catenin, an adherens junction component and key Wnt pathway effector, regulates numerous developmental processes and supports embryonic stem cell (ESC) pluripotency in specific contexts. The β-catenin homologue γ-catenin (also known as Plakoglobin) is a constituent of desmosomes and adherens junctions and may participate in Wnt signaling in certain situations. Here, we use β-catenin^(+/+)^ and β-catenin^(−/−)^ mouse embryonic stem cells (mESCs) to investigate the role of γ-catenin in Wnt signaling and mESC differentiation. Although γ-catenin protein is markedly stabilized upon inhibition or ablation of GSK-3 in wild-type (WT) mESCs, efficient silencing of its expression in these cells does not affect β-catenin/TCF target gene activation after Wnt pathway stimulation. Nonetheless, knocking down γ-catenin expression in WT mESCs appears to promote their exit from pluripotency in short-term differentiation assays. In β-catenin^(−/−)^ mESCs, GSK-3 inhibition does not detectably alter cytosolic γ-catenin levels and does not activate TCF target genes. Intriguingly, β-catenin/TCF target genes are induced in β-catenin^(−/−)^ mESCs overexpressing stabilized γ-catenin and the ability of these genes to be activated upon GSK-3 inhibition is partially restored when wild-type γ-catenin is overexpressed in these cells. This suggests that a critical threshold level of total catenin expression must be attained before there is sufficient signaling-competent γ-catenin available to respond to GSK-3 inhibition and to regulate target genes as a consequence. WT mESCs stably overexpressing γ-catenin exhibit robust Wnt pathway activation and display a block in tri-lineage differentiation that largely mimics that observed upon overexpression of β-catenin. However, β-catenin overexpression appears to be more effective than γ-catenin overexpression in sustaining the retention of markers of naïve pluripotency in cells that have been subjected to differentiation-inducing conditions. Collectively, our study reveals a function for γ-catenin in the regulation of mESC differentiation and has implications for human cancers in which γ-catenin is mutated and/or aberrantly expressed.

## Introduction

β-catenin is an Armadillo domain-containing protein with roles in both cell-cell adhesion, through interactions with cell surface cadherin molecules [1], and in the highly conserved Wnt pathway, which regulates a multitude of biological processes throughout embryonic development and thereafter [2]–[4]. The core components of the Wnt pathway include glycogen synthase kinase-3 (GSK-3), Axin1/2 and Adenomatous Polyposis Coli (APC), which serve to limit the cellular levels of β-catenin [3]. In the unstimulated state, β-catenin is N-terminally phosphorylated by GSK-3, destining it for proteasomal degradation [4]. By this mechanism, the cytoplasmic and nuclear levels of β-catenin are kept low. After pathway stimulation, cytoplasmic β-catenin levels rise, and once a threshold is exceeded, it enters the nucleus to transactivate target genes through binding to TCF/LEF transcription factors [5]. Elevated β-catenin levels are observed in many human cancers due to mutations in one or more Wnt/β-catenin pathway components [6]. While the rheostatic activation and deactivation of Wnt signaling plays critical roles throughout development, inappropriate, persistent activation of Wnt signaling promotes oncogenesis.

There is accumulating evidence that the Wnt pathway plays a critical role in the regulation of embryonic stem cell (ESC) properties (reviewed recently in [2]). Stimulation of the Wnt pathway has been correlated with enhanced ESC pluripotency and has been shown to facilitate induced pluripotent stem cell (iPSC) generation and the derivation of ESC lines from refractory mouse strains [7]–[15]. Indeed, Wnt3a and LIF in basal media are sufficient to maintain mESCs in the highest level of pluripotency, known as naïve pluripotency, with Wnt3a serving to prevent differentiation to the “primed” pluripotent state that is observed in epiblast stem cells (EpiSCs) [15]. In support of a role for β-catenin in pluripotency maintenance, our laboratory demonstrated that mouse ESCs (mESCs) lacking GSK-3, which exhibit highly elevated β-catenin levels and hyperactivation of the Wnt pathway, are extremely refractory to neuronal differentiation [16]. Intriguingly, this effect appears to be independent of prototypical β-catenin-mediated activation of TCF signaling, but rather may involve the interaction of β-catenin with the core pluripotency regulator Oct-4, as well as β-catenin's de-repression of TCF3-mediated transcriptional repression [9], [17]–[19].

Originally identified as a component of desmosomes [20], the β-catenin homologue, γ-catenin (also known as Plakoglobin), was thereafter found to associate with adherens junctions, where its binding to cadherins is mutually exclusive to that of β-catenin [21]–[24]. Aside from these structural roles, multiple lines of evidence suggest that γ-catenin can regulate the Wnt pathway, including: (1) Similar to β-catenin, γ-catenin overexpression induces a duplicate axis phenotype in *Xenopus laevis* [25], [26]; (2) γ-catenin co-immunoprecipitates with APC and Axin [27]–[29]; (3) Upstream regulators of Wnt signaling, including Wnt1 ligand, APC and Axin, regulate γ-catenin stability [27], [30], [31]; and (4) γ-catenin can transactivate β-catenin/TCF reporter constructs [32]–[35]. However, due to the fact that γ-catenin is unable to rescue the embryonic lethality of β-catenin^(−/−)^ mice [36], [37], additional studies are warranted to further clarify the role of γ-catenin in the Wnt pathway.

In this study, we utilized β-catenin^(+/+)^ and β-catenin^(−/−)^ mESCs [38] to further elucidate the role of γ-catenin in Wnt signaling and in the regulation of mESC differentiation. We find that although γ-catenin is robustly stabilized upon Wnt pathway stimulation in mESCs, suppressing its expression has no detectable impact on the activation of Wnt reporters or target genes. Moreover, despite its compensatory upregulation in β-catenin^(−/−)^ mESCs, in these cells γ-catenin is not stabilized by GSK-3 inhibition; as a result, it cannot substitute for β-catenin as an effector of Wnt signaling in this context. However, ectopic expression of γ-catenin in WT mESCs robustly activates Wnt target genes, blocks neuronal differentiation and reinforces the retention of pluripotent stem cell markers Oct-4, Sox2 and Nanog, whereas knocking down its expression via shRNAs appears to promote exit from the pluripotent state. The overexpression of γ-catenin does not appear to be as potent as the overexpression of β-catenin in preventing differentiation to an EpiSC-like state, as assessed by marker analyses in short term differentiation assays. Intriguingly, ectopic expression of γ-catenin in β-catenin^(−/−)^ cells is sufficient to enable TCF target gene activation upon the inhibition of GSK-3, suggesting that a threshold level of γ-catenin is required before it is accessible for regulation by the machinery of the β-catenin destruction complex. Collectively, our findings suggest partial functional overlap between γ-catenin and β-catenin in the regulation of mESC properties, and have implications for cancers in which γ-catenin is mutated and/or misexpressed.

## Materials and Methods

### Cell culture

Cell culture supplements were obtained from Gibco (Life Technologies) unless otherwise specified. Mouse ESCs (β-cat^+/+^) were maintained in DMEM (Thermo) supplemented with 15% FBS (Fisher), 1× nonessential amino acids, 1× L-glutamine, 1× sodium pyruvate, 55 µM β-mercaptoethanol (Sigma; M7522) and 1000 U/ml ESGRO® LIF (Millipore). All cells were kept in a humidified incubator at 37°C, 5% CO_2_. The medium used for EB experiments was identical to that above, except that it contained 5% FBS and lacked LIF. The N2B27 culture medium used to initiate neural differentiation consisted of a 1∶1 ratio of Neurobasal media and DMEM/F12 with N2 and B27 supplements [39].

### Antibodies

The following antibodies were used for western blotting and/or immunofluorescent staining: mouse anti-γ-catenin/Plakoglobin (610253, BD Transduction); mouse anti-β-catenin (610153, BD Transduction); rabbit anti-β-catenin (9587, Cell Signaling Tech.); mouse anti-FLAG-tag (F1804, Sigma); mouse anti-GAPDH (ab8245, Abcam); mouse anti-β-Tubulin 1 (T7816, Sigma); mouse anti-Myc-tag (4A6, Upstate); rabbit anti-Nanog (A300-397A, Bethyl Laboratories); mouse anti-Oct3/4 (sc-5279, Santa Cruz); rabbit anti-Sox2 (2748, Cell Signaling Tech.); mouse anti-β-III-Tubulin (MAB1195, R&D Systems).

### Plasmids

To generate myc-tagged γ-catenin expression constructs in pCAG-IP [40], the human γ-catenin cDNAs were PCR-amplified using pcDNA-γ-catenin (wild-type, S28A and ΔC [34]) as template, and the resulting products were inserted into pCR®-Blunt-II-TOPO® (Invitrogen). The cDNAs were then excised using EcoRI and ligated into pCAG-IP. To generate pCAG-IP-γ-catS28AΔC, the region encompassing the S28A mutation was transferred from pCAG-IP-γ-catS28A into pCAG-IP-γ-catΔC using XbaI and BglII. All plasmids were verified by sequencing (Mobix, McMaster University).

### Cell lysate preparation

All chemicals were purchased from Sigma, unless otherwise specified. To prepare whole cell lysates, 60 mm dishes of cells were rinsed twice with PBS at room temperature and were then lysed with ice-cold RIPA buffer [150 mM NaCl, 1% NP-40, 0.5% DOC, 0.1% SDS, 50 mM Tris pH 8.0, 1 mM EDTA, and Halt Protease Inhibitor Cocktail (Thermo)] on ice for 10 minutes. Lysed cells were transferred into 1.5 mL tubes on ice and centrifuged at 16, 100 × g for 10 minutes (4°C). The supernatants, containing soluble proteins, were isolated and the protein content was quantified using the Lowry method (DC Protein Assay; Bio-Rad). The samples were then prepared for electrophoresis by diluting them to contain 1× LDS buffer (Invitrogen) with 5% TCEP Bond-Breaker solution (Thermo) and heating them for 5 minutes at 95°C prior to western blot analysis. Hypotonic (cytosolic) lysates were prepared by lysing PBS-washed cells in ice-cold 1 × hypotonic lysis buffer (50 mM Tris pH 7.4, 1 mM EDTA, and 1× Halt Protease Inhibitor Cocktail), incubating on ice for 30 minutes, and then centrifuging at 235, 000 × g in a Sorvall M150 ultracentrifuge for 30 minutes at 4°C. The supernatant (cytosol) was collected and the protein content was quantitated as described above.

### Nuclear and cytoplasmic extraction

Protein extracts from nuclear and cytoplasmic fractions were performed as described previously [41]. In brief, 60 mm dishes of cells were washed twice with PBS and were then scraped off the plates and pelleted at 16, 100 × g for 15 minutes (4°C). The supernatant was then discarded and the cell pellet was resuspended in Tween-20 lysis buffer [25 mM Tris/Hepes pH 8.0, 50 mM NaCl, 2 mM EDTA, 1 mM phenylmethylsulfonyl fluoride (PMSF), 0.5% Tween-20, and 1× Halt Protease Inhibitors (Thermo)] and incubated on ice for 10 minutes. Samples were then centrifuged at 6, 000 × g for 1 minute at 4°C to isolate nuclear (pellet) and cytoplasmic (supernatant) fractions. The pellet was further resuspended in Tween-20 lysis buffer containing 500 mM NaCl and incubated on ice for 15 minutes. Additional Tween-20 lysis buffer (lacking NaCl) was added directly to each sample prior to centrifuging at 16, 100 × g for 15 minutes to recover the supernatant containing the purified nuclear fraction.

### shRNA-mediated knockdown of γ-catenin expression

HuSH 29mer shRNA constructs against *Jup* were purchased from Origene (Cat. No. TR501141). After a preliminary screen to identify the most effective shRNA sequence in mESCs, an shRNA construct harbouring the following sequence was used in these studies: γ-catSH: AGAGTGGCAACAGACATACACCTACGACT. The negative control constructs used were: 1) Vector (empty pRS vector); and 2) NegCtrlSH (non-effective shRNA control designed by Origene, Cat No. TR30012).

### Stable cell line generation

Transgenic and knockdown cell line generation using mESCs was performed as described previously [9]. Briefly, the β-cateninS33A line was generated by linearizing pCAGGS-βcatS33A-IRES-Puro and transfecting this construct into mESCs, prior to selection using puromycin (2 µg/ml) for 7 days. Clones were then isolated, expanded, and characterized. The γ-cateninΔC, γ-cateninS28A, and γ-cateninS28AΔC stable cell lines were made in a similar manner. Parental wild-type (β-catenin^(+/+)^) and β-catenin^(−/−)^ mESC cell lines were derived in Dr. Austin Cooney's laboratory, and have been described previously [38].

### TCF reporter assays

mESCs were cotransfected with the constructs 8X TOPFlash (1.8 µg), driving firefly luciferase [42], and pRL-CMV (0.2 µg), driving expression of renilla luciferase for normalization (Promega). Cells were washed twice with PBS 24 hours following transfection, and lysed with passive lysis buffer (Promega). The luciferase reporter activities were measured using a luminometer as per the manufacturer's instructions (Promega Dual-Light System).

### Quantitative RT-PCR

Total RNA was isolated using the PureLink RNA Mini Kit (Life Technology), and 1 µg was used to generate cDNA with SuperScript II reverse transcriptase (Invitrogen). The cDNA was diluted to 100 µl and 3 µl of this was used for each 20 µl PCR reaction with PerfeCTa® SYBR Green® FastMix®, ROX™ (Quanta Biosciences). β-actin was used as the housekeeping gene for all qRT-PCR assays. Stratagene's Mx3000P instrument and accompanying MxPro software or Bio-Rad's CFX96 instrument and CFX Manager™ software were used to determine relative gene expression levels using the delta-delta Ct method with data obtained after 40 cycles of PCR. Primer sequences were designed using IDT's online primer design software (idtdna.com) or were obtained from prior publications and are listed in Table 1.

**Table 1:** Quantitative RT-PCR Primers.

Primer	Sequence	Reference
Actin (β) FWD	5′-TTGCTGACAGGATGCAGAAGGAGA-3′	[9]
Actin (β) REV	5′-ACTCCTGCTTGCTGATCCACATCT-3′	[9]
Axin2 FWD	5′-AAAGAAACTGGCAAGTGTCCACGC-3′	[9]
Axin2 REV	5′-GGCAAATTCGTCACTCGCCTTCTT-3′	[9]
Brachyury FWD	5′-AGCTCTCCAACCTATGCGGACAAT-3′	[9]
Brachyury REV	5′-TGGTACCATTGCTCACAGACCAGA-3′	[9]
Cardiac Troponin FWD	5′-GTAGAGGACACCAAACCCAAG-3′	N/A
Cardiac Troponin REV	5′-GAGTCTGTAGCTCATTCAGGTC-3′	N/A
β-catenin FWD	5′-TGCCTTCAGATCTTAGCTTATGG-3′	[9]
β-catenin REV	5′-AGACAGCACCTTCAGCAC-3′	[9]
γ-catenin FWD	5′-GTCCTGTTCCGCATCTCTG-3′	N/A
γ-catenin REV	5′-TCTGCATAGGGTTCGTTGATC-3′	N/A
Cdx1 FWD	5′-AGAGCGGCAGGTAAAGATCTGGTT-3′	[9]
Cdx1 REV	5′-AGAAGGCCAGCATTAGTAGGGCAT-3′	[9]
Esrrb FWD	5′-CATGAAATGCCTCAAAGTGGG-3′	N/A
Esrrb REV	5′-AAATCGGCAGGTTCAGGTAG-3′	N/A
Fgf5 FWD	5′-AATATTTGCTGTGTCTCAGG-3′	[15]
Fgf5 REV	5′-TAAATTTGGCACTTGCATGG-3′	[15]
α-fetoprotein FWD	5′-GATGAAACCTATGCCCCTCC-3′	N/A
α-fetoprotein REV	5′-CTGTCAGTTCAGGCTTTTGC-3′	N/A
Klf4 FWD	5′-ACTTGTGACTATGCAGGCTG-3′	N/A
Klf4 REV	5-ACAGTGGTAAGGTTTCTCGC-3′	N/A
Map2 FWD	5′-CTGGATTTCAAGGAAAAGGCC-3′	[9]
Map2 REV	5′-ATCTCAGCCCCGTGATCTA-3′	[9]
c-Myc FWD	5′-GCTGTTTGAAGGCTGGATTTC-3′	[9]
c-Myc REV	5′-GATGAAATAGGGCTGTACGGAG-3′	[9]
Nanog FWD	5′-AACCAAAGGATGAAGTGCAAGCGG-3′	[9]
Nanog REV	5′-TCCAAGTTGGGTTGGTCCAAGTCT-3′	[9]
Nestin FWD	5′-AAGTTCCCAGGCTTCTCTTG-3′	[9]
Nestin REV	5′-GTCTCAAGGGTATTAGGCAAGG-3′	[9]
Otx2 FWD	5′-CATGATGTCTTATCTAAAGCAACCG-3′	[15]
Otx2 REV	5′-GTCGAGCTGTGCCCTAGTA-3′	[15]
Pecam-1 FWD	5′-CAAAGTGGAATCAAACCGTATCT-3′	[15]
Pecam-1 REV	5′-CTACAGGTGTGCCCGAG-3′	[15]
Rex1 FWD	5′-GCTCCTGCACACAGAAGAAA-3′	[15]
Rex1 REV	5′-GTCTTAGCTGCTTCCTTCTTGA-3′	[15]
Stella FWD	5′-TTCAAAGCGCCTTTCCCAA-3′	[15]
Stella REV	5′-ACATCTGAATGGCTCACTG-3′	[15]
β-III-Tubulin FWD	5′-CGCCTTTGGACACCTATTCAG-3′	[9]
β-III-Tubulin REV	5′-TTCTCACACTCTTTCCGCAC-3′	[9]
Tyrosine Hydroxylase FWD	5′-AAGATCAAACCTACCAGCCG-3′	[9]
Tyrosine Hydroxylase REV	5′-TACGGGTCAAACTTCACAGAG-3′	[9]

### Embryoid Body (EB) formation and immunofluorescent staining of EBs

EB formation and staining were performed as described previously [9]. In brief, the indicated cell lines were grown on inactivated mouse embryonic fibroblasts (iMEFS) for 24 hours, pre-plated on gelatin-coated dishes to selectively remove the iMEFs, counted with a Countess® automated cell counter (Invitrogen) and plated in EB media (5% FBS, -LIF) on ultra-low attachment 6-well dishes (Corning). At the time of lysis, the EBs were collected and washed twice with PBS by centrifuging for 5 minutes at 270 × g prior to adding the appropriate lysis buffer. To prepare for staining, EBs were harvested, washed twice with PBS, and fixed overnight in 4% paraformaldehyde/PBS at 4°C. The fixed EBs were then incubated twice in 0.2% Triton X-100/PBS for 15 minutes at room temperature (RT) and subsequently kept at 4°C in 2% BSA/PBS (blocking solution) for 2 hours. Primary and secondary antibodies were diluted with blocking solution. EBs were then incubated with anti-β-III-Tubulin (1∶500) at 4°C on a rotator overnight. The EBs were then rinsed 3 times in PBS the next day (rotating for 15 minutes at RT for each wash), prior to incubating in secondary antibody (1∶1000) on a rotator at 4°C overnight. On day five, the EBs were washed five times with PBS and subsequently mounted on slides in ProLong Gold antifade reagent with DAPI (Invitrogen). Afterwards, the EBs were imaged using an Olympus IX-81 epifluorescence microscope with a DSU spinning disk confocal imaging attachment.

### Alkaline phosphatase staining

Cell lines were grown on iMEFs for 24 hours, prior to pre-plating to remove the iMEFs, as described above. Cells were subsequently counted and plated in the various conditions indicated. After 72 hours, cells were fixed in 4% paraformaldehyde for 2 minutes, washed in 0.2% TBS-T and incubated in AP staining reagent (Fast Red Violet Solution:Napthol:H_2_O in a 2∶1∶1 ratio; Millipore, SCR004) for 15 minutes at room temperature in the dark. An additional wash with 0.2% TBS-T was performed before the cells were visualized using an Olympus IX-81 microscope and standard brightfield illumination.

### Statistical Analyses

Analysis of variance (ANOVA) and unpaired t-test analyses were performed using the statistical software package Prism (GraphPad).

## Results

### Stabilization of γ-catenin in mESCs after inhibition/ablation of GSK-3

**Figure 1:**
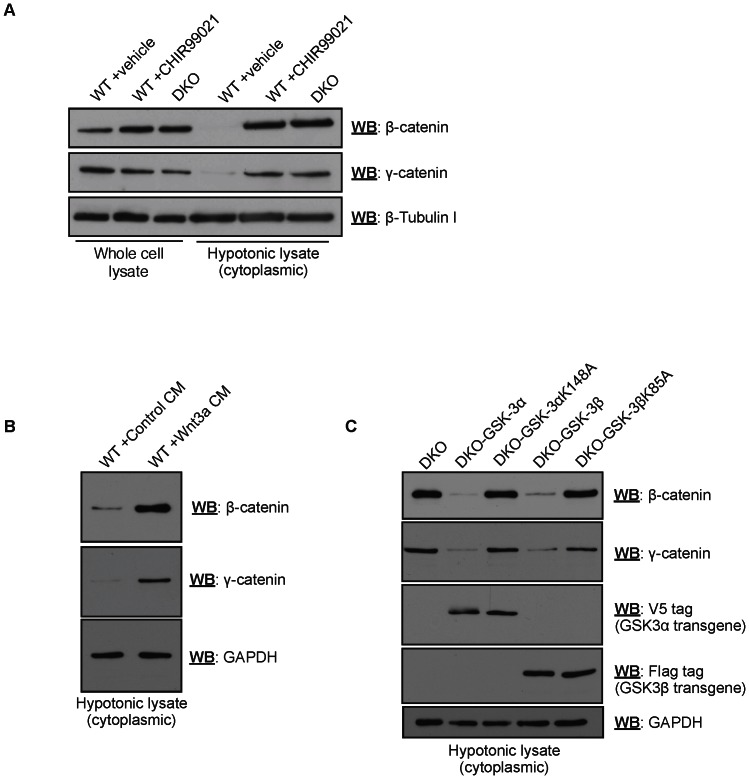
Cytosolic γ-catenin is stabilized in response to GSK-3 inhibition/ablation in wild-type mESCs. (A) Robust cytosolic stabilization of β-catenin and γ-catenin in mESCs after treatment with CHIR99021 (15 µM for ∼24 hours), and in mESCs lacking both isoforms (α and β) of GSK-3 (DKO), as assessed by western blotting of hypotonic lysates. Increases in β-catenin and γ-catenin levels were not clearly observed using whole cell lysates for western blotting purposes after the same treatment. (B) β-catenin and γ-catenin stabilization in mESCs after Wnt pathway activation using Wnt3a-conditioned medium (∼24 hours). (C) β-catenin and γ-catenin stabilization in DKO mESCs is reversed by re-expression of either wild-type GSK-3α or GSK-3β, but not their kinase-dead forms (K148A and K85A, respectively).

**Figure 2:**
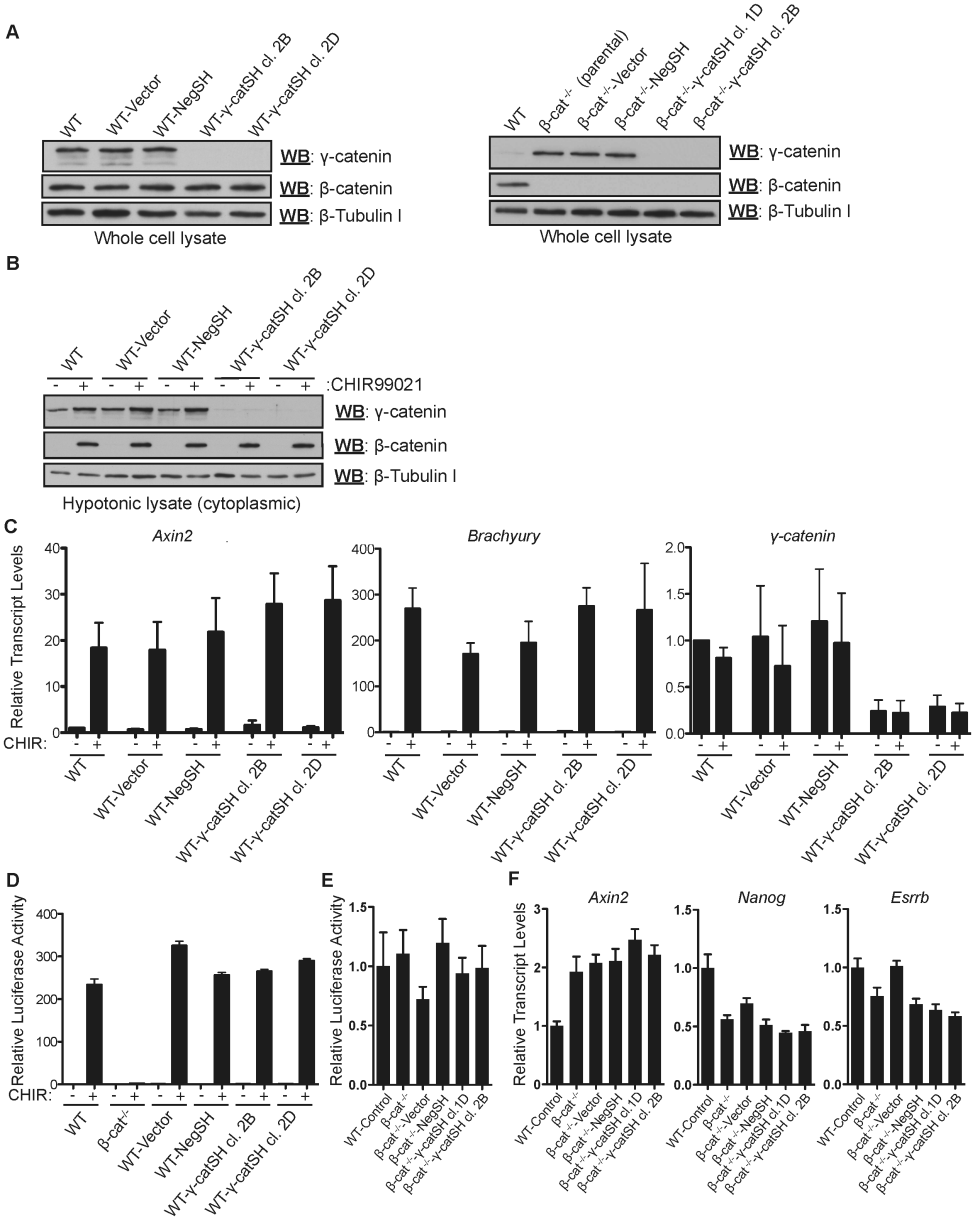
γ-catenin knockdown does not influence β-catenin/TCF target gene transactivation in WT and β-cat^(−/−)^ mESCs. (A) Stable γ-catenin knockdown lines were generated in WT and β-cat^(−/−)^ mESCs. Clones were isolated, expanded and assayed for γ-catenin suppression by western blotting using whole cell extracts. γ-catenin knockdown in most clones was highly efficient and β-catenin levels were unaltered by the γ-catenin-specific shRNAs in WT mESCs. (B) γ-catenin cytoplasmic accumulation is severely compromised in γ-catenin knockdown lines after GSK-3 inhibition (15 µM for ∼24 hours). (C and D) The activation of prototypical Wnt/TCF target genes (*Axin2, Brachyury*), or a TCF reporter construct, is not impeded by stable γ-catenin knockdown. Bars represent means and error bars denote s.d. (n = 2). (E) Stable γ-catenin knockdown lines were generated in β-catenin^(−/−)^ mESCs. Clones were isolated, expanded and assayed for TCF reporter activity and (F) activation of the Wnt/TCF target gene *Axin2*, as well as pluripotency markers *Nanog* and *Esrrb* via qRT-PCR analysis. Wnt activity remains unaltered when γ-catenin is suppressed in the absence of β-catenin. Bars represent means and error bars indicate s.e.m (n = 3).

Although previous studies have linked γ-catenin to the regulation of Wnt signaling by virtue of its homology to β-catenin, few have directly assayed whether γ-catenin stability is regulated by GSK-3-mediated phosphorylation. To determine whether γ-catenin was stabilized in mESCs after inhibition of GSK-3, we treated cells with the highly specific GSK-3 inhibitor CHIR99021 (15 µM for 24 hours), and assayed for cytoplasmic stabilization of γ-catenin using western blotting (Fig. 1). As expected, β-catenin was robustly stabilized after GSK-3 inhibition (Fig 1A), treatment with Wnt3a-conditioned medium (Fig. 1B), or in mESCs lacking GSK-3 (DKO) (Fig. 1A,C). Importantly, we reproducibly observed robust γ-catenin stabilization after treatment with CHIR99021, Wnt3a-conditioned medium, or in DKO mESCs. γ-catenin stabilization in DKO mESCs could be reversed by the single copy, site-specific, stable re-expression of wild-type GSK-3α or GSK-3β, but not their kinase dead forms (K148A and K85A, respectively; Fig. 1C). The observed increase in protein levels was not due to increased β-/γ-catenin transcription, consistent with previous observations (Fig. 2C and data not shown). Of note, stabilization of β-catenin or γ-catenin after GSK-3 inhibition was rarely, if ever, detected in mESC whole-cell extracts (Fig. 1A); this observation was likely attributable to signal saturation by highly abundant, junctional β-/γ-catenin molecules. Collectively, these experiments suggested that γ-catenin levels are regulated in a manner that is reminiscent to that of β-catenin, and that γ-catenin may contribute to the transactivation of Wnt target genes.

### Knockdown of γ-catenin does not impede activation of the Wnt pathway

To address whether endogenous γ-catenin participates in the activation of β-catenin/TCF target genes in mESCs, we generated cell lines in which expression of γ-catenin was stably suppressed using shRNAs (Fig. 2). We isolated and characterized multiple γ-catenin knockdown clones in wild-type and β-catenin-null genetic backgrounds; the efficiency of knockdown for most clones was very high, as determined by western blotting (Fig. 2A). We had no difficulty obtaining stable β-catenin^(−/−)^ cell lines with efficent γ-catenin knockdown. In wild-type mESCs, compensatory upregulation of β-catenin was not observed in γ-catenin knockdown clones (Fig. 2A). We assayed whether γ-catenin knockdown in wild-type mESCs influenced the activation of an 8X-SuperTopflash β-catenin/TCF reporter, as well as established Wnt target genes (*Axin2* and *Brachyury*), in response to GSK-3 inhibition (15 µM for ∼24 hours). No readily detectable stabilized γ-catenin was observed in cytoplasmic extracts derived from γ-catenin knockdown lines (clones 2B and 2D) after treatment with CHIR99021, relative to controls (Fig. 2B). As expected, *Axin2* and *Brachyury* were robustly upregulated in WT and control lines (Vector and NegSH) after treatment with CHIR99021 (Fig. 2C). Importantly, γ-catenin knockdown, though very efficient, did not impede the activation of *Axin2* or *Brachyury* expression (Fig. 2C), or a β-catenin/TCF reporter (Fig. 2D), after GSK-3 inhibition. Additionally, knockdown of γ-catenin in β-catenin^(−/−)^ mESCs did not significantly (as determined by ANOVA analyses) reduce basal TCF reporter activity or the expression of the general TCF target gene *Axin2* or the pluripotency-related TCF3 target genes *Nanog* and *Esrrb* (Fig. 2E,F). These data strongly support the conclusion that endogenous γ-catenin does not contribute, in any appreciable/detectable way, to the activation of β-catenin/TCF target genes in WT mESCs with inhibited GSK-3 activity or to the resting level of TCF target gene expression in mESCs lacking β-catenin.

### γ-catenin does not substitute for β-catenin in the Wnt pathway in β-catenin^(−/−)^ mESCs

**Figure 3:**
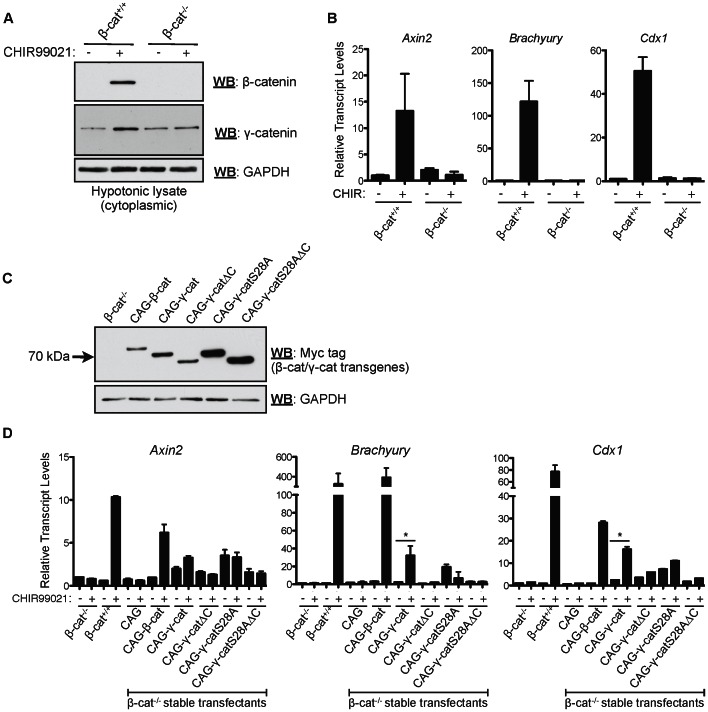
Ectopic expression of γ-catenin in β-cat^(−/−)^ cells rescues TCF target gene activation upon GSK-3 inhibition. (A) Cytosolic γ-catenin is not stabilized after GSK-3 inhibition in β-cat^(−/−)^ mESCs. (B) Established β-catenin/TCF target genes (*Axin2, Brachyury and Cdx1*) are not induced by GSK-3 inhibition in β-cat^(−/−)^ mESCs, indicating that γ-catenin does not substitute for β-catenin in this context. Bars represent means and error bars indicate s.e.m. (n = 3) (C) Myc-tagged β-catenin, and variants of γ-catenin, were stably expressed under the control of the CAG promoter in β-cat^(−/−)^ mESCs, and clones were isolated, expanded and screened for transgene expression by western blotting. (D) Ectopic, stable expression of γ-catenin partially rescues the transactivation of β-catenin/TCF target genes (*Axin2*, *Brachyury* and *Cdx1*) in β-cat^(−/−)^ mESCs after GSK-3 inhibition. Bars represent means and error bars indicate s.e.m. (n = 3). Asterisks indicate p<0.05 in unpaired t-test analyses between indicated groups.

Although γ-catenin knockdown had no detectable effect on Wnt signaling in WT mESCs (Fig. 2), we hypothesized that it may compensate for β-catenin deficiency in β-catenin^(−/−)^ mESCs (Fig. 3). Indeed, γ-catenin was upregulated in whole cell extracts from β-catenin^(−/−)^ mESCs (compare lanes 1 and 2 of Fig. 2A, right panel), consistent with similar observations in β-catenin-null mice [36], [37]. To our surprise, however, cytoplasmic levels of γ-catenin were unchanged after GSK-3 inhibition in β-catenin^(−/−)^ mESCs (Fig. 3A). Accordingly, GSK-3 inhibition did not result in the upregulation of *Axin2*, *Brachury* or *Cdx1* transcripts in β-catenin^(−/−)^ mESCs (Fig. 3B). We conclude that endogenous γ-catenin is unable to substitute for β-catenin in the Wnt pathway in β-catenin^(−/−)^ mESCs.

One possible explanation for this observation is that, in the absence of β-catenin, limiting amounts of endogenous γ-catenin are preferentially targeted to desmosomes and/or adherens junctions, leaving none in the pool reserved for signaling purposes. To account for this possibility, we increased the cellular γ-catenin levels by ectopically expressing myc-tagged wild-type and mutant forms of γ-catenin in β-catenin^(−/−)^ mESCs (Fig. 3C), and asked whether these forms were capable of transducing Wnt signals (Fig. 3D). As a control, we re-expressed myc-tagged wild-type β-catenin in β-catenin^(−/−)^ mESCs; as expected, these cells exhibited elevated *Axin2*, *Brachyury* and *Cdx1* expression following GSK-3 inhibition (Fig. 3D). Intriguingly, ectopically expressed wild-type myc-γ-catenin was capable of activating the expression of *Axin2*, *Brachyury* and *Cdx1*, albeit less efficiently than β-catenin (Fig. 3D). This activation was further enhanced by GSK-3 inhibition. β-catenin^(−/−)^ mESCs expressing a mutant form of γ-catenin, lacking its C-terminal TCF transactivation domain (γ-catΔC), remained largely incapable of Wnt target gene activation, even after GSK-3 inhibition. γ-catS28A, harboring a serine to alanine point mutation at a putative GSK-3 phosphorylation site [43], induced elevated basal *Axin2*, *Brachyury* and *Cdx1* expression in the β-catenin^(−/−)^ mESCs in the presence or absence of GSK-3 inhibitor. The ability of γ-catS28A to induce target gene expression required an intact C-terminus, as γ-catS28AΔC-expressing cell lines displayed negligible target gene activation (Fig. 3D).

### Stable expression of γ-catenin enhances retention of pluripotency marker expression and prevents the neuronal differentiation of mESCs

**Figure 4:**
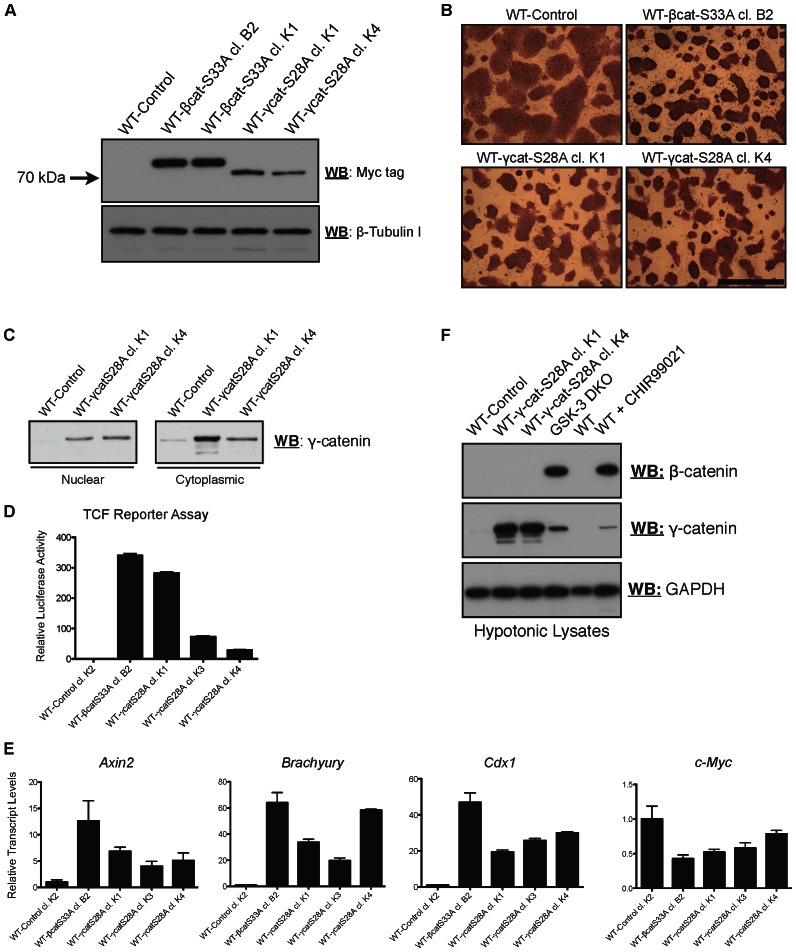
Ectopic expression of γ-cateninS28A in mESCs activates the Wnt pathway without altering cytosolic β-catenin levels. (A) mESCs were stably transfected with a Myc-tagged S28A variant of γ-catenin. Clones were isolated, expanded and screened for γ-catS28A expression by western blotting of hypotonic lysates using a tag-specific antibody. (B) Alkaline phosphatase staining of WT, γ-catS28A-expressing and β-catS33A-expressing mESC colonies, visualized using bright-field microscopy. Bar  = 400 µm. (C) Western blot analyses of nuclear and cytosolic extracts prepared from WT and γ-catS28A cell lines, as indicated. Elevated levels of γ-cat protein were detected in both cytoplasmic and nuclear lysates of γ-catS28A-overexpressing cell lines. (D) TCF reporter activity of mESCs stably expressing γ-catS28A. (E) Induction of the β-catenin/TCF target genes, *Axin2, Brachyury* and *Cdx1*, but not *c-Myc*, in mESCs stably expressing γ-catS28A or β-catS33A. (F) Expression of stabilized γ-catenin in WT mESCs does not result in detectable levels of cytosolic β-catenin as determined by western blot analysis of hypotonic lysates. Cytosolic levels of β-catenin in GSK-3 DKO mESCs or WT mESCs treated with CHIR99021 (24 h, 15 µM) are provided as positive controls. For (D) and (E), Bars represent means and error bars indicate s.e.m. (n = 3).

It has become increasingly clear that the inhibition of GSK-3 enhances the self-renewal of pluripotent mESCs in culture, and facilitates the derivation of ESC lines from resistant strains from different species [11], [12], [14]. Several recent studies suggest that β-catenin is likely the primary mediator of these effects [9], [17], [18], [39], [44], [45]. mESCs expressing a stabilized mutant of β-catenin (S33A), exhibit hyperactivation of Wnt target genes, enhanced self-renewal, and are extremely refractory to differentiation [9], [45]. It is unknown whether these abilities are specific to β-catenin, or extend to γ-catenin. To this end, we generated mESC lines stably expressing the S28A variant of γ-catenin (γ-catS28A; Fig. 4). Ectopically expressed γ-catS28A accumulated to detectable levels in the cytoplasm of transfected cells (Fig. 4A). γ-catS28A clones exhibited a morphology that was reminiscent of β-catS33A clones [9] and displayed high levels of alkaline phosphatase activity as determined by using colorimetric staining (Fig. 4B). Western blot analyses of nuclear and cytosolic fractions obtained from wild-type and γ-catS28A-expressing mESC lines revealed that cytosolic accumulation of γ-catS28A reflected nuclear accumulation (Fig. 4C). Transgenic γ-catS28A-expressing mESCs exhibited varying levels of TCF reporter activity and β-catenin/TCF target gene activation (Fig.4D,E), which in general, were less than those observed in mESCs expressing β-catS33A. Interestingly, TCF reporter assay results for the γ-catS28A-overexpressing clones did not always correlate well with the target gene expression results for the same clones (e.g. reporter activity for clone K4 was very low, whereas *Brachyury* expression was similar to that obtained with stable β-catenin overexpression), suggesting that the ability of γ-catenin to activate gene expression is dependent on the context of target sequences.

It has been reported that overexpression of γ-catenin can be associated with concomitant increases in stabilized β-catenin in some tumor cell types [34], [46]. In mESCs, we find that stable expression of γ-catS28A does not result in increased levels of cytosolic β-catenin, as detected by western blotting (Fig. 4F). This suggests that the TCF reporter and “β-catenin” target gene activation we observe in mESCs overexpressing γ-catS28A likely does not involve co-stabilized β-catenin. Thus, the mechanism through which overexpressed γ-catenin exerts its effects on target genes appears to differ depending on the nature of the cell models being examined.

**Figure 5:**
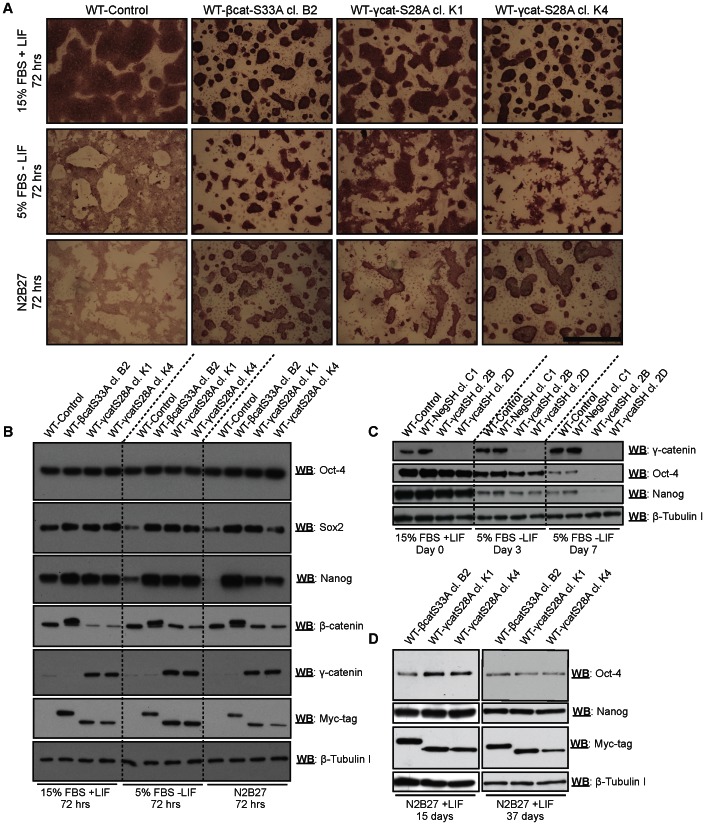
Ectopic γ-catenin expression delays, whereas endogenous γ-catenin knockdown accelerates, loss of pluripotency in differentiating mESCs. (A and B) The indicated mESC lines were seeded into standard ES medium or media conducive to differentiation, either N2B27 (lacking serum and LIF), or DMEM with 5% FBS and lacking LIF (EB medium), and assayed after 72 hours for β-galactosidase activity and the expression of pluripotency markers (Oct-4, Nanog and Sox2) by western blot analysis. (A) mESC lines expressing either β-catS33A or γ-catS28A exhibited a highly compact colony morphology and retained high levels of alkaline phosphatase staining after 72 hours of differentiation. Bar  = 400 µm. (B) These lines also retained the expression of Nanog and Sox2, whereas the control line (WT-Control) lost expression of these markers as assessed by western blotting. (C) Bulk EBs were generated using γ-catenin wild-type and knockdown lines by culturing 2×10^6^ cells on low attachment dishes with 5% FBS and lacking LIF. Western blot analysis using whole cell extracts taken on days 0, 3 and 7 revealed that wild-type and control (NegSH) lines retained the expression of Oct-4 and Nanog more effectively than γ-catenin knockdown lines. (D) mESCs stably expressing β-catS33A or γ-catS28A were seeded into N2B27 medium with LIF, and cultured as loosely adherent spheres, with passaging every 3–4 days over prolonged culture. The expression of Oct-4 and Nanog protein was readily detected in western blots of whole cell lysates from these stable cell lines.

We next examined the differentiation capabilities of γ-catS28A mESCs. To make an initial assessment of the ability of γ-catS28A mESCs to exit the pluripotent state, we used short-term loss of pluripotency assays, essentially as were performed previously for mESCs expressing β-catS33A [9]. We seeded mESCs harboring either β-catS33A or γ-catS28A (clones K1 and K4) into medium with 5% FBS and lacking LIF (commonly used for the generation of embryoid bodies), or N2B27 medium lacking serum and LIF. After 72 hours, the cells were stained for alkaline phosphatase activity, and imaged or subjected to western blot analyses to assay for the retention of pluripotency markers (Oct-4, Sox2 and Nanog; Fig. 5). Whereas the control line (WT-Control) exhibited a flatter morphology and barely detectable alkaline phosphatase staining after 72 hours of differentiation in either 5% FBS (without LIF) or N2B27, both the β-catS33A and γ-catS28A lines maintained a more compact morphology and displayed clear alkaline phosphatase staining when subjected to the same conditions (Fig. 5A). Western blot analyses using β-catS33A and γ-catS28A lysates revealed that both lines maintained the expression of the pluripotency markers Sox2 and Nanog, while the control line displayed dramatically reduced expression of these factors after being subjected to the differentiation-inducing conditions (Fig. 5B). We then assessed the capability of endogenous γ-catenin to assist in the retention of the expression of pluripotency markers over the course of a 7-day bulk EB differentiation assay. Using γ-catenin knockdown cell extracts, western blot analyses revealed that knockdown lines lost the expression of Oct-4 and Nanog more rapidly than wild-type and negative-control shRNA-expressing cell lines (Fig. 5C). Collectively, these experiments suggest that the expression of stabilized γ-catenin is sufficient to reinforce the more primitive, pluripotent state of mESCs, even under conditions permissive of differentiation and that endogenous γ-catenin may contribute to stabilizing the pluripotent state in WT mESCs.

Although we demonstrated that ectopic expression of β-catS33A and γ-catS28A delays the loss of pluripotency in N2B27 medium (Fig. 5A,B), we found that these lines cannot be cultured indefinitely in this medium, as they succumb to differentiation and death after 3–4 passages. However, β-catS33A and γ-catS28A mESCs were amenable to culture as loosely adherent spheres on gelatin-coated dishes in N2B27 medium supplemented with LIF, for prolonged periods of time (several weeks), while maintaining expression of pluripotency markers (Fig. 5D). Thus, expression of γ-catS28A circumvents the requirement for serum, which is typically required for mESC propagation in LIF-containing culture medium.

**Figure 6:**
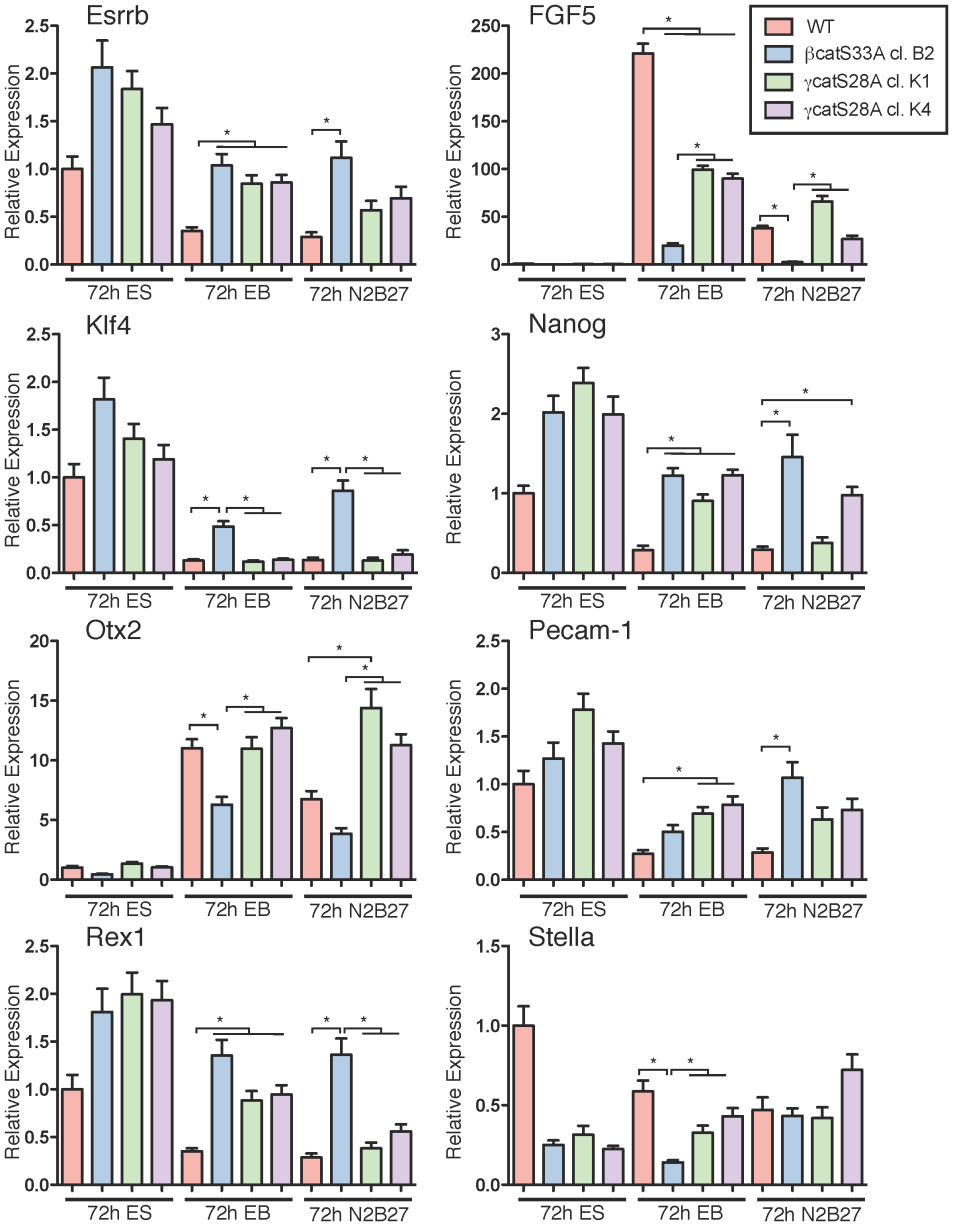
Overexpressed stabilized γ-catenin is less efficient than overexpressed stabilized β-catenin in sustaining retention of markers of ground state pluripotency in WT mESCs induced to differentiate. The indicated mESC cell lines were maintained for 72 hours using conditions identical to those described in Fig. 5A, namely, incubation in ES, EB or N2B27 media. Six markers of embryonic stem cells: *Esrrb*, *Klf4*, *Nanog*, *Pecam-1*, *Rex1* and *Stella* and two markers of EpiSCs: *FGF5* and *Otx2*, were assessed by qRT-PCR analyses. Data are presented in 8 individual graphs, where the transcript level for each gene is shown relative to that detected in wild-type cells maintained for 72 hours in standard ES medium (mean value arbitrarily set at 1). Bars represent the mean values of three experimental replicates and error bars indicate s.e.m. Asterisks indicate statistically significant differences (p<0.05) between the indicated means as determined by ANOVA analyses with Tukey's multiple comparison post-tests, which were applied to the gene expression datasets from the EB and N2B27 experiments.

As it has been reported that Wnt/β-catenin signaling serves to prevent the differentiation of naïve pluripotent mESCs to primed pluripotent mEpiSCs [15], we were interested in determining if β-catenin and γ-catenin overexpression had the same effect on mESC differentiation towards an EpiSC-like state. Using the same cell lines and differentiation conditions described in Fig. 5A, we examined the mRNA expression of ESC markers *Esrrb*, *Klf4*, *Nanog*, *Pecam-1*, *Rex1* and *Stella* [47]–[50] and EpiSC markers *FGF5* and *Otx2* [15], [51], by using qRT-PCR analyses (Fig. 6). In standard ES medium (15% FBS + LIF), overexpression of either stabilized β-catenin or stabilized γ-catenin resulted in modestly elevated (approximately 2-fold or less) transcript levels for the mESC markers *Esrrb*, *Klf4*, *Nanog*, *Pecam-1* and *Rex1*, compared to the levels detected in WT control mESCs. By contrast, *Stella* expression was approximately 4-fold lower in β-catS33A and γ-catS28A ovexpressing mESCs than in WT mESCs, which was unexpected. Taken together, with the exception of *Stella*, the marker analyses indicate that mESCs overexpressing β-catS33A and γ-catS28A, when maintained in LIF-containing medium, reside in a state of naïve pluripotency. The unexpected *Stella* results suggest that this gene does not share the same regulatory controls that guide the expression of the other core pluripotency factors in response to Wnt/β-catenin signaling.

Wild-type mESCs maintained in EB differentiation conditions for 72 hours (5% FBS, no LIF) showed dramatically upregulated expression of the EpiSC marker *FGF5* (more than 200-fold higher expression in EB conditions relative to ES conditions). In the same differentiation conditions, mESCs overexpressing γ-catS28A displayed attenuated expression of *FGF5* compared to the expression observed in WT mESCs (approx. 50% of WT levels) but overexpression of β-catS33A was much more efficient in suppressing *FGF5* expression in cells maintained in EB medium (approx. 10% of WT levels). With regard to the EpiSC marker *Otx2*, γ-catS28A overexpression had no effect on the induction of *Otx2* expression in EB culture conditions. That is, cell lines overexpressing γ-catS28A displayed an approximately 10-fold higher level of *Otx2* expression in EB relative to ES conditions, which was also observed in the WT cells. By contrast, mESCs overexpressing β-catS33A showed levels of *Otx2* expression in EB conditions that were approximately 50% of those detected in WT mESCs.

In EB differentiation conditions, WT mESCs displayed a reduction in the expression of mESC markers, *Esrrb*, *Nanog*, *Pecam-1* and *Rex1* to approximately 25% of the expression observed in ES medium, whereas the cell lines overexpressing stabilized β-catenin or stabilized γ-catenin displayed expression of these markers at levels similar to those obtained from analyses of WT mESCs in ES medium. The expression of *Klf4*, a marker of naïve pluripotent cells, was differentially affected by the expression of stabilized β-catenin versus stabilized γ-catenin. Cells expressing γ-catS28A displayed the same reduction in *Klf4* expression as observed in WT mESCs in EB medium, whereas β-catS33A-expressing cells retained approximately 5-fold higher levels of *Klf4* expression in the same conditions. In the N2B27 differentiation conditions, which promote neurectodermal differentiation [39], β-catS33A mESCs expressed levels of the pluripotency markers *Esrrb*, *Klf4*, *Nanog*, *Pecam*-*1* and *Rex1*, which were the same or higher than those detected in WT mESCs maintained in ES medium. By contrast, the expression of these markers was reduced to similar extents in WT mESCs and γ-catS28A mESCs maintained in N2B27 medium. Taken together, our data suggest that β-catenin overexpression is more effective than γ-catenin overexpression in sustaining the retention of markers of naοve pluripotency, and preventing EpiSC differentiation, in cells that have been subjected to differentiation-inducing conditions.

**Figure 7:**
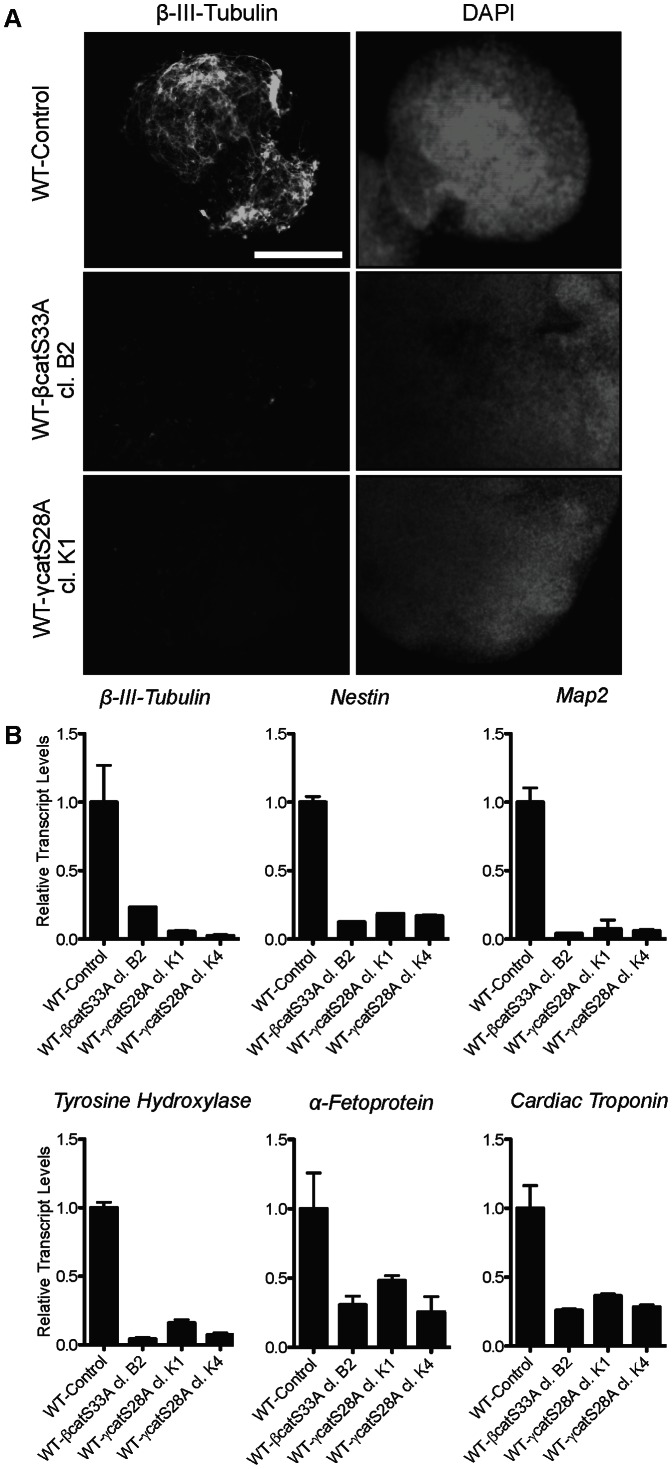
Stabilized γ-catenin suppresses neuronal differentiation in embryoid body assays and reduces overall tri-lineage differentiation efficiency. (A and B) Embryoid bodies were generated using control, β-catS33A, and γ-catS28A mESCs, and assayed for neuronal differentiation after 10 days. (A) Only WT EBs displayed immunofluorescent staining for the neuronal marker, β-III-tubulin. (B) The transcript levels of the neuronal markers *β-III-tubulin*, *Nestin*, *Map2*, *Tyrosine hydroxylase*, the endoderm marker *α-Fetoprotein*, and the mesoderm marker *Cardiac Troponin*, were reduced in EBs derived from mESCs overexpressing β-catS33A or γ-catS28A, as assessed by qRT-PCR analyses. Bars represent means and error bars indicate s.e.m. (n = 2). Size bar  = 200 µm.

To assess the tri-lineage differentiation capacities of β-catS33A and γ-catS28A mESCs, we generated embryoid bodies using these lines, and examined the levels of neuronal markers by immunofluorescence (Fig. 7A) and qRT-PCR analysis (Fig. 7B) after 10 days of differentiation. Similar to β-catS33A EBs, γ-catS28A EBs were markedly larger than EBs generated from control mESCs (data not shown). In contrast to control EBs, γ-catS28A EBs expressed negligible amounts of the neuronal marker β-III-tubulin, as determined by immunofluorescent staining of whole EBs (Fig. 7A). Consistent with this observation, qRT-PCR analyses revealed significantly reduced expression of several neuronal markers in β-catS33A and γ-catS28A EBs, relative to control EBs, as well as less dramatically reduced expression of α-fetoprotein and cardiac troponin, markers of endoderm and mesoderm differentiation, respectively (Fig. 7B). These data indicate that stable expression of γ-catS28A, like β-catS33A, renders mESCs highly refractory to neuronal differentiation and reduces their overall tri-lineage differentiation ability. However, our data suggest that β-catenin overexpression is more effective than γ-catenin overexpression in sustaining the retention of markers of naïve pluripotency and preventing EpiSC differentiation in cells that have been subjected to differentiation-inducing conditions.

## Discussion

Since its original discovery as a component of desmosomal plaques, through the use of a novel monoclonal antibody [20], it has become clear that γ-catenin resides at multiple subcellular localizations, including the plasma membrane, cytoplasm, and nucleus, where it executes seemingly disparate biological functions (reviewed in [52]). Owing to its structural and functional similarities to β-catenin, including its ability to induce the characteristic duplicate axis phenotype when overexpressed in *Xenopus laevis* embryos [25], γ-catenin had been proposed to participate in Wnt signaling, likely by competing or cooperating with β-catenin in the transactivation of β-catenin/TCF target genes. Most of the analyses of γ-catenin function in the Wnt pathway have been undertaken using *Xenopus laevis* or immortalized mammalian cell lines as model systems. In our study, we used mESCs as a physiologically relevant cell culture model that more accurately reflects the signaling events in early mammalian development. In mESCs, we found that, similar to β-catenin, γ-catenin is robustly stabilized upon Wnt pathway stimulation, achieved either through small molecule-mediated inhibition of GSK-3, or through treatment with Wnt3a-conditioned medium (Fig. 1). This is consistent with, and expands upon, the findings of previous studies demonstrating that Axin/γ-catenin complexes regulate γ-catenin stability *in vitro* [27], and that Wnt-1 increases soluble γ-catenin levels in two immortalized cancer cell lines [30].

We used stably expressed γ-catenin-specific shRNAs to demonstrate that, in mESCs, suppression of γ-catenin expression does not detectably affect the transactivation of β-catenin/TCF target genes in response to Wnt pathway stimulation via GSK-3 inhibition (Fig. 2). This experiment suggests that, under “normal” developmental conditions, γ-catenin is unlikely to be a meaningful effector of Wnt signaling, and that in this setting, highly abundant β-catenin molecules underlie the majority of Wnt pathway signaling. One might have expected that in β-catenin^(−/−)^ cells, γ-catenin would become stabilized in response to GSK-3 inhibition and thereby substitute for β-catenin's role in the activation of TCF target genes. However, consistent with a recent study [44], our data indicates that this does not occur (Fig. 3). Instead, although the steady-state γ-catenin levels are higher in β-cat^(−/−)^ mESCs, we found that GSK-3 inhibition in these cells does not elicit the cytoplasmic stabilization of γ-catenin, suggesting that β-catenin is needed to somehow maintain and/or enhance γ-catenin stability in response to pathway activation. Various possibilities exist as to why this might be the case. Perhaps β-catenin is an imperative and irreplaceable component of the pathway, and its presence is needed to recruit γ-catenin to the β-catenin destruction complex, where its stability can be modulated by pathway activation. Alternatively, any cellular γ-catenin in β-cat^(−/−)^ mESCs may be fully sequestered by desmosomes and adherens junctions, such that the critical signaling pool cannot be regulated via the destruction complex *per se*. Our findings with ectopically expressed γ-catenin in β-cat^(−/−)^ mESCs support this second scenario, as γ-catenin overexpression partially rescues the ability of β-cat^(−/−)^ cells to respond to GSK-3 inhibition by activating TCF target genes (Fig. 3D). Thus, in the complete absence of β-catenin, γ-catenin can function as a GSK-3-regulated transcriptional transactivator if its steady-state levels are augmented. The question remains whether the findings herein can be extended to most or all other cell types, and whether, under conditions of moderate β-catenin levels (instead of the ‘all or none’ scenario of β-cat^(+/+)^ and β-cat^(−/−)^ mESCs), might γ-catenin then contribute to the transactivation of Wnt target genes?

A previous study by Shimizu and colleagues assessed the roles of β-catenin and γ-catenin in Wnt signaling using the murine embryonal carcinoma (EC) F9 line [33]. By using homologous recombination, the genes encoding β-catenin and γ-catenin were ablated separately and in combination, and the effects on Wnt signaling were assessed. Although our study and that of Shimizu *et al*. ultimately reach similar conclusions regarding the role of endogenous γ-catenin in Wnt signaling, our studies differ in certain key ways. First, the F9 line employed by Shimizu *et al*. harbors chromosomal abnormalities [53], some of which may affect intracellular signaling cascades and sensitivity to ligands (e.g. Wnts). In contrast to our experiments using mESCs, in which we detect robust cytosolic γ-catenin stabilization after treatment with Wnt3a-conditioned medium (Fig. 1B), Shimizu *et al*. observed only very modest γ-catenin stabilization, suggesting that the Wnt pathway does not appreciably affect γ-catenin stability in this cell line. Furthermore, our studies are the first to describe the functional consequences of γ-catenin overexpression and knockdown in mESCs with respect to their differentiation capacities in short-term assays.

In a small but significant number of human cancers, γ-catenin protein becomes mislocalized and/or its levels become elevated [43], [46], [54]–[57]. Morgan *et al*. recently showed that γ-catenin is frequently overexpressed and mislocalized to the nucleus in acute myeloid leukemia [46]. Based on their findings, they suggested that γ-catenin might synergize with nuclear β-catenin activity, amplifying transcription of target genes. In mESCs, γ-catenin overexpression does not appear to alter the stability of cytosolic β-catenin (Fig. 4F), suggesting that in this system, synergism with β-catenin is not a requirement for target gene activation. Interestingly, in NCI-H28 cells, which lack β-catenin expression, γ-catenin has been shown to upregulate the TCF target gene *Survivin* [57], which is in keeping with our finding that ectopic expression of γ-catenin in β-cat^(−/−)^ mESCs rescues their ability to regulate TCF target genes. Kolligs *et al*. [31] found that γ-catenin possesses transforming activity in a rat kidney epithelial cell line in which γ-catenin strongly activates *c-myc* expression, whereas β-catenin does not. We directly interrogated whether a form of γ-catenin, analogous to that harboring the S28A missense mutation identified by Caca *et al*. [43], could transactivate a TCF reporter construct and established Wnt target genes (*Axin2*, *Brachyury*, *Cdx1*) when stably expressed in mESCs. Indeed, this form of γ-catenin robustly activated the TCF reporter and Wnt target genes (Fig. 4D,E), supporting the hypothesis that it may play an important role in the pathogenesis of cancers in which its expression becomes elevated. We did not observe a significant increase in *c-Myc* transcript levels in WT mESCs overexpressing stabilized β-catenin or γ-catenin; indeed, slightly decreased *c-Myc* expression was detected (Fig. 4E). This suggests that the regulation of target genes by γ-catenin is likely dependent on the cellular context.

Interestingly, previous studies have suggested that γ-catenin variants harboring the S28A mutation do not exhibit significantly enhanced stability relative to wild-type γ-catenin, which is itself inherently more stable than β-catenin [35]. We found that γ-catS28A transactivated β-catenin/TCF target genes slightly more efficiently than wild-type γ-catenin when ectopically expressed in β-cat^(−/−)^ mESCs (Fig. 3D). As proposed by others [35], this may explain why “activating” γ-catenin mutations (e.g. S28A) are rarely detected in human cancers. Nonetheless, it is worth noting that according to the Wellcome Trust Sanger Institute COSMIC database (http://www.sanger.ac.uk/re-sources/databases/cosmic.html), mutations in the gene encoding γ-catenin (*Jup*) occur in a small proportion of lung, ovarian and breast cancers. Whether these mutations give rise to forms of γ-catenin with altered signaling activity and/or expression patterns, remains to be determined.

There is accumulating evidence that small molecule-mediated inhibition of GSK-3, or activation of Wnt/β-catenin signaling by other means, reinforces mESC self-renewal and enhances iPSC derivation [9]–[19], [38], [45], [49]. Through the combined efforts of various groups, it is now apparent that β-catenin is a key mediator of these effects [2], [9], [17]–[19], [44], [45]. Several recent studies have proposed mechanisms through which β-catenin regulates the pluripotent state. Our laboratory demonstrated that β-catenin interacts with the core pluripotency regulator, Oct-4, thereby enhancing its activity and reinforcing self-renewal [9]. A recent study has revealed that β-catenin and Oct-4 interact in membrane-associated complexes, which correlate with ground state pluripotency in mESCs [19]. Other studies have shown that β-catenin derepresses the activity of TCF3 [17], [18], an established transcriptional repressor of *Nanog*, a key target of which, *Esrrb*, has been linked to the effects of GSK-3 inhibition in mESCs [48], [49]. Our current study suggests that similar to β-catenin, γ-catenin overexpression can potentiate the transcription of markers of the pluripotent ground state when mESCs are maintained in medium containing LIF (Fig. 6). In conditions that promote mESC differentiation though, β-catenin overexpression is much more efficient than γ-catenin overexpression at sustaining the expression of markers of naïve pluripotency (Fig. 6). Thus, mESCs overexpressing stabilized γ-catenin appear to be susceptible to differentiating into an EpiSC-like state, unlike mESCs overexpressing stabilized β-catenin. Nonetheless, overexpression of both β-catenin and γ-catenin in mESCs effectively blocks their efficient tri-lineage differentiation capacity, especially towards the neurectodermal lineage.

The precise mechanisms through which overexpressed γ-catenin exerts different effects from overexpressed β-catenin on mESC pluripotency and differentiation remain to be elucidated. It has been previously reported that γ-catenin is less efficient than β-catenin in forming a ternary complex with LEF1 and the LEF1-binding sequence [58]. Thus, less efficient complexing of γ-catenin with the TCF/LEFs may underlie the diminished ability of γ-catenin overexpression, compared with β-catenin overexpression, to sustain pluripotent marker expression in mESCs. Another possibility is that GSK-3-mediated regulation of γ-catenin stability may influence desmosomal structure and/or signaling. Considering that γ-catenin has emerged as an indispensable regulator of desmosome assembly, reviewed in [59], perhaps its stabilization or destabilization due to alterations in GSK-3 activity would affect intercellular adhesion. It is worth noting that loss of desmosome-based adhesion has been implicated in the acceleration of epithelial-mesenchymal transition (EMT) and enhanced tumor metastasis [60]. Whether the regulation of γ-catenin through GSK-3 and/or Wnt signaling might affect such processes requires further investigation.

Overall, our study has demonstrated that the β-catenin homologue, γ-catenin, is not an integral component of the Wnt signaling cascade in mESCs, nor can endogenous γ-catenin substitute for β-catenin in this pathway of β-catenin^(−/−)^ mESCs. However, in WT mESCs, overexpression of γ-catenin is sufficient to activate Wnt signaling without a requirement for β-catenin stabilization and knocking down endogenous γ-catenin levels appears to accelerate the exit of WT mESCs from the pluripotent state, suggesting that γ-catenin may aid in the maintenance of pluripotency. Our data also show that γ-catenin is responsive to GSK-3 inhibition, and can activate TCF target genes when the levels of its expression reach a critical threshold. Although β-catenin and γ-catenin both appear to function similarly when overexpressed in mESCs, based on their ability to block neurectodermal differentiation in embryoid body assays, there are clear differences in the ability of these proteins to sustain the expression of pluripotency markers and to prevent ESC-to-EpiSC differentiation. Taken together, our data suggest a mechanism through which γ-catenin may play a role in the genesis of cancers in which it becomes mutated and/or misexpressed, whereby it may promote the sustenance of a more primitive and/or de-differentiated cellular state.
